# Dynamic Output Feedback and Neural Network Control of a Non-Holonomic Mobile Robot

**DOI:** 10.3390/s23156875

**Published:** 2023-08-03

**Authors:** Manuel Cardona, Fernando E. Serrano

**Affiliations:** Research Department, Universidad Don Bosco, San Salvador 1874, El Salvador; manuel.cardona@udb.edu.sv

**Keywords:** mobile robot, non-holonomy, driftless control

## Abstract

This paper presents the design and synthesis of a dynamic output feedback neural network controller for a non-holonomic mobile robot. First, the dynamic model of a non-holonomic mobile robot is presented, in which these constraints are considered for the mathematical derivation of a feasible representation of this kind of robot. Then, two control strategies are provided based on kinematic control for this kind of robot. The first control strategy is based on driftless control; this means that considering that the velocity vector of the mobile robot is orthogonal to its restriction, a dynamic output feedback and neural network controller is designed so that the control action would be zero only when the velocity of the mobile robot is zero. The Lyapunov stability theorem is implemented in order to find a suitable control law. Then, another control strategy is designed for trajectory-tracking purposes, in which similar to the driftless controller, a kinematic control scheme is provided that is suitable to implement in more sophisticated hardware. In both control strategies, a dynamic control law is provided along with a feedforward neural network controller, so in this way, by the Lyapunov theory, the stability and convergence to the origin of the mobile robot position coordinates are ensured. Finally, two numerical experiments are presented in order to validate the theoretical results synthesized in this research study. Discussions and conclusions are provided in order to analyze the results found in this research study.

## 1. Introduction

Mobile robots have been widely implemented since their introduction several decades ago due to the vast applications of these kinds of robots. It is important to mention also that mobile robots have been implemented for several tasks in military and civil missions. For these reasons, it is important to design and synthesize several kinds of control strategies for these robots, taking into consideration the imperatives for trajectory-tracking, path following, leader–follower missions, etc. Due to the simplicity of the mobile robot dynamics and their implementation in hardware platforms, it is important to remark that kinematic control is abundant for these kinds of robots. The control strategies for robots are diverse; among these control strategies are the strategies based on robust control, sliding mode control, fuzzy control, and neural control, among others. Restriction in the dynamics of mobile robots which are found commonly are of non-holonomic type. These kinds of restrictions are based considering the kinematic and dynamics properties of the mobile robot. That is why these restrictions provide a way to develop driftless control strategies in order to provide an easy way to implement these control approaches.

Taking into consideration that the dynamic modeling of mobile robots is very important to this research study, it is crucial to mention the following research papers in which this topic is considered. So, for example, in papers like [1], the dynamic modeling with its uncertainties is shown. Then, in [2], a non-holonomic wheeled mobile robot with unknown dynamics is represented mathematically for control purposes. Then, in [3], a non-holonomic mobile robot is modeled with unknown dynamics. In [4], a remarkable paper which is very important for this research study, the researchers analyze the dynamics of a lightweight mobile robot for longitudinal motion. Meanwhile, in [5], the dynamic modeling and sliding mode control of a tractor mobile robot is presented. Then, in [6], a complete book chapter about the dynamic modeling of mobile robots is presented considering the longitudinal and lateral slip. All these research studies consider the dynamic modeling of a mobile robot. However, it is important to mention that while in general, the dynamics of mobile robots is quite simple, sometimes, in order to obtain the mathematical representation of a mobile robot’s dynamics, it is important to consider unmodeled and unknown dynamics. The studies the literature have yet to clarify that the dynamic controllers and neural controllers are sufficiently robust for kinds of mobile robots.

Holonomic constraints are crucial to mention in this research study, taking into consideration that these kinds of constraints, especially the non-holonomic constraints, are found in mobile robots. These constraints are related to the velocity vector field of the mobile robot, and the mathematical explanations of these kind of constraints are mentioned in the content of this research paper. For these reasons, it is important to mention the following research papers found in the literature. For example, in papers like [7], non-holonomic constraints for geometric control theory are explained. Meanwhile, in [8], an interesting research paper about the singularities of holonomic and non-holonomic robotic systems is presented. In [9], the researchers studied holonomic and non-holonomic deformations in the AB equations, which are useful for atmospheric fluid modeling. Then, in [10], the dynamical invariant-based quantum gates are presented. In [11,12], researchers presented the model predictive control of a holonomic mobile robot and an adaptive robust controller for mechanical systems with non-holonomic trajectories, respectively.

A mobile robot’s dynamic model constraints allow us a way to obtain efficient control strategies by the driftless control method. The driftless control method consists of obtaining a zero control effort only if the velocity of the mobile robot is equal to zero. In the literature, there are different driftless control techniques for different kinds of robots or mechanisms, so for example, in [13], the calculation of the control effort of two input driftless control systems is presented. Then, in [14], the switched driftless control of a kind of non-holonomic system is shown. Meanwhile, in [15], a driftless oscillation control for a nonlinear systems is provided. Then, in [16], the researchers studied the controllability of a driftless nonlinear time-delayed system. In [17,18], the involutive flows of a nonlinear driftless control system and the asymptotic control for wheeled mobile robot with driftless constraints are evinced, respectively.

One of the most important theoretical fundamentals for this research study is the design and implementation of dynamic output feedback controllers, taking into consideration that a hybrid control strategy based on dynamic output feedback control is implemented for these kinds of mobile robots. In papers like [19], a dynamic output feedback control for a switched affine system based on L−∞ control is presented. It is important to mention [20,21], in which the dynamic output feedback control of a networked control system is presented and a mixed dynamic output feedback control for an active suspension system with actuator saturation and time delays are presented, respectively. It is important to mention that in the second paper, a hybrid control strategy with dynamic output feedback and fuzzy type-2 controllers is implemented, taking into consideration that this control strategy provides an optimal framework for the present research study. In [22], the dynamic output feedback control of a Luré system is proposed. Then, in [23,24], the consensus of a linear multi-agent system by reduced order dynamic output feedback and the robust stabilization for an uncertain singular Markovian-jump system via dynamic output feedback control is achieved.

It is important to mention that neural control is a suitable control strategy taking into consideration that a hybrid control strategy for a mobile non-holonomic robot is presented. For example, in [25], a neural fault-tolerant controller with input constraints for an output manipulator with output constraints is presented. Meanwhile, in [26], indirect neural control for an unmanned surface vessel is presented considering injection and deception attacks. Then, in [27,28], a quasi-optimal neural control for solar thermal systems and neural-based fixed optimal control for the attitude tracking of a space vehicle with output constraints are evinced, respectively. Finally, in [29,30], a space manipulator neural output constrained control for a space manipulator using a Lyapunov tan-barrier functional and the neural network control of nuclear plants are evinced, respectively.

It is also important to mention two phenomena found in practice in mobile robotics regarding the control, stabilization and trajectory tracking of mobile robots in the presence of input saturation and time delays. It is important to consider the following references regarding the trajectory tracking control of mobile robots with input saturation. For example, in [31], the adaptive stabilization and control of mobile robots with input saturation is shown. This paper is important for this present research study considering that non-holonomic constraints are included in the development of the control strategy. In this paper, input saturation is not considered because of the design of driftless and non-driftless control approaches, which are novel. In [32], the input saturation is considered for the control allocation of a mobile robot. Then, in [33], the visual tracking of a mobile robot is performed with the saturated inputs of velocity and acceleration. In [34], the robust control tracking design of a mobile robot with input saturation is presented.

The time-delay phenomenon is found in many kinds of linear and nonlinear dynamic systems. Mobile robots are not the exception in which time delays are found. It is important to mention the following references regarding this topic. For example, in [35], the control synchronization of mobile robots with input time delays is evinced. Meanwhile, in [36], the control of mobile robots with time delays is presented. Then, in [37], a predictive control for mobile robots with time delays is presented. Finally, in [38], the control of mobile robots with time-varying delays and noise attenuation is shown.

It is good to clarify that all the numerical simulations were performed in GNU Octave 4.2.2. The implementation of multibody dynamics commercial software or a real-time experimental setup will be implemented as a future direction of this research study. Despite this, it is important to mention the following references regarding the multibody dynamics simulation implementing commercial software. It is found in references like [39], where an 8 × 8 vehicle is simulated by the implementation of multibody dynamics commercial software. Meanwhile, in [40], a railway vehicle is simulated by the implementation of the previous mentioned commercial software. Finally, in [41,42], the researchers explained other multibody analyses for different kinds of mechanisms or vehicles.

This paper presents the implementation of a hybrid control strategy which consists of a neural and dynamic output feedback controller for a non-holonomic mobile robot. We show two control strategies: the first one is a driftless control system based on the dynamic output feedback and neural controller, and the second one is a full controller based also on neural and feedback output control. The dynamic output feedback and neural control are designed implementing a Lyapunov functional suitable to obtain the control law in both cases. The neural network implemented in this research study is a feedforward neural network in order to facilitate the controller design. The two control strategies ensure the stability for trajectory tracking purposes and the convergence to zero for the error variable, which comprise the difference between the desired trajectory and the measured trajectory. It is important to mention that two numerical experiments are provided in order to validate the theoretical results of this research study. Discussion and conclusions are provided at the end of this research study.

It is important to remark that the main contribution of this research study is that non-holonomy is one of the complexities found in some kinds of mobile and robotics systems. So, for this reason, this research study provides two control approaches related to non-holonomy in mobile robotic systems. The first approach consists of a driftless controller that is simple and easy to implement in specific in available hardware in many research laboratories in the world. The driftless control strategy provides the easiness of being implemented in mobile robotics due to its compactness and low computational effort. In addition, the driftless control strategy provides the important characteristic that when the control input is zero, the mobile robot’s velocity is zero. Meanwhile, the non driftless control strategy is more adequate when a robust and compact strategy is necessary for the trajectory tracking of the mobile robot. The neural networks are tuned offline, so the implementation of this control strategy is straightforward due to the implementation in real-time hardware only requiring matricial and vectorial operations, making the controller adequate for a more viable control strategy for these kinds of mobile robots.

It is important to mention that the neural controller component of this hybrid control strategy comprises a standard feedforward neural network. The difference between the neural network implemented in this research study and the neural network used for comparison purposes is that the latest one is tuned and implemented standalone in comparison with the dynamic and neural network controller. It is important to mention that the neural network implemented for comparison purposes is marginally stable in comparison with the neural network which comprises the proposed hybrid control strategy, which is asymptotically stable.

The strengths of the proposed control strategy rely on the improvements of the performance in comparison that other control strategies found in the literature, such as PID control, sliding mode control and neural network control when these strategies are implemented standalone in comparison to when these strategies are implemented as a hybrid control strategy. It is important to mention that the theoretical results are validated numerically, corroborating that this control strategy surpasses other control strategies found in the literature. It is worthwhile to mention that one advantage of the proposed control strategy is that the neural network controller is tuned offline in order to obtain the optimal performance; meanwhile, as long as the neural network controller component meets the stability results, the closed-loop stability is ensured. It is worthwhile to mention that the dynamic controller part along with the neural controller part improves the closed-loop performance in comparison with other control techniques. Finally, it is observed that a novelty of this research study is that the proposed controller is designed for driftless and non-driftless control.

## 2. Related Work

In this section, some related work that is worthwhile to mention in this research study is presented. This literature review consists of the following items related to non-holonomic mobile robots and their control:

- Kinematics of mobile robots.
- Non-holonomic mobile robots.
- Dynamic output feedback of mobile robots.
- Neural control of mobile robots.
- Miscellaneous control strategies for mobile robots.

It is important to mention the following references related to the kinematics of mobile robots because they are crucial for this research study. For example, in [43], a kinematic Lyapunov-based controller is presented for mobile robots. Then, in [44], a kinematic-based control strategy for a spherical mobile robot driven by a 2D pendulum is shown. Meanwhile, in [45], a singularity free kinematic model of a degenerated mobile robot is obtained. Meanwhile, in the following references [46,47,48], the kinematic control and two kinematic models of mobile robots are presented.

In this section, it is important to mention the following research study taking into consideration that a kinematic and dynamic model design for non-holonomic mobile robots is important for this research study. Among these research studies, we found the following. In [49], the distance-based control of a non-holomic mobile robot swarm is presented. Meanwhile, in [50], the motion and force control of mobile robots is presented by means of a fuzzy wavelet neural network controller. Then, in [51], the distributed formation control for a swarm of mobile robots is presented, taking into consideration the velocity constraints and iterative learning. Meanwhile, in [11,52], a model predictive path following the control strategy for holonomic mobile robots and the real-time identification of different types of non-holonomic mobile robots are presented, respectively. In [53], the trajectory tracking of a non-holonomic mobile robot is presented by means of sliding mode control with disturbances.

Dynamic output feedback control has been implemented specifically for mobile robots. For this reason, it is important to mention the following control strategies, which are found in the literature considering the importance that they have for this research study. In [54,55], the researchers mentioned the cooperative output control of a mobile flexible manipulator and also the distributed output feedback control of non-holonomic mobile robots with only the leader’s position measurement. Then, in [56,57], an output trajectory tracking of mobile robots and an adaptive tracking control by means of output feedback for mobile robots are presented, respectively. Then, in [58], an output tracking of a non-holonomic mobile robot with fractional order visual feedback is evinced. In [59], a finite-time output feedback tracking control of a non-holonomic mobile robot is presented.

It is important to mention in this research study some neural robot control strategies which are found in the literature. So, for example, in papers like [60], a vision approach with deep neural networks to control autonomous mobile robots is presented. Meanwhile, in [61], the kinematics of a cable-driven parallel robot is achieved by the implementation of neural networks. Then, in [62], the trajectory tracking of a self-balancing robot by adaptive neural networks is performed. Then, in [63], a fault diagnosis for the harmonic reducer of industrial robots is achieved by means of neural networks. Other examples of the implementation of neural networks are found in [64,65] in which in the first case, the integrated consensus control of multi-robots using neural model predictive control is outlined. Meanwhile, in the second reference, a neural control for a robotic manipulator with an input deadzone is presented.

To finalize this section, the following references are related to miscellaneous robot control strategies related in any other way to mobile robot control. In papers like [66,67], model predictive and model control are implemented for the control of different kinds of robots. Then, in [68,69], two control strategies are presented for the trajectory tracking of mobile robots. Finally, in [11,70], model predictive and adaptive full state constrained tracking control for mobile robots are presented, respectively.

## 3. Notation

In this section, the notations used in this research study are presented in order to facilitate the paper’s readiness and clarify the theoretical results obtained in this research study. All the operators used in this research study are evinced in this section in order to elucidate the mathematical background used.

1. 〈.,.〉 is the inner product defined in a Hilbert space.
2. $\left[f,g\right]=\frac{\partial g}{\partial x}f-\frac{\partial f}{\partial x}g$ is the Lie bracket.
3. $\frac{\partial f}{\partial x}=\left(\begin{array}{ccc} \frac{\partial f_{1}}{\partial x_{1}} & \ldots & \frac{\partial f_{1}}{\partial x_{n}}\\ \vdots & \ddots & \vdots \\ \frac{\partial f_{n}}{\partial x_{1}} & \ldots & \frac{\partial f_{n}}{\partial x_{n}} \end{array}\right)$ is the Jacobian of a vector field.
4. ∥.∥ is the 2-norm defined in a Euclidean space.

## 4. Problem Formulation

In this section, the kinematic model of the mobile robot is presented. It is important to mention that only a generic mobile robot is implemented in this research study. The intention is to drive the robot according to a pre-specified trajectory. The kinematics of the mobile robot is given by the following equations:

(1) $$\begin{array}{ccc} sin\left(\theta \right)\dot{x}-cos\left(\theta \right)\dot{y} & = & 0\\ sin\left(\theta +\phi \right)\dot{x}-cos\left(\theta +\phi \right)\dot{y} & = & 0 \end{array}$$

**Figure 1:**
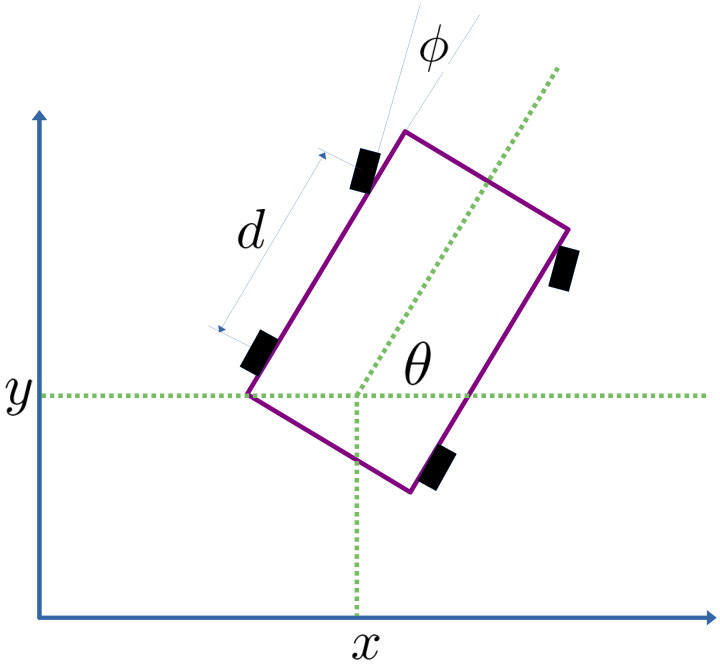
Schematic drawing of the mobile robot used in this research study.

As can be noticed in Figure 1, the coordinates and mobile robot length are defined as shown in the figure. In order to facilitate the mathematical tractability of this kinematic model in order to obtain the proposed control strategies, the following equivalent kinematic model is obtained, as shown in [71]. Consider the following change of variable $q={\left[x,y,\theta ,\phi \right]}^{T}$, so (1) can be obtained as:

(2) $$\begin{array}{ccc} \langle \omega_{1},\dot{q}\rangle & = & \left[\begin{array}{cccc} sin(\theta ) & cos(\theta ) & 0 & 0 \end{array}\right]\dot{q}=0\\ \langle \omega_{2},\dot{q}\rangle & = & \left[\begin{array}{cccc} sin(\theta +\phi ) & -cos(\theta +\phi ) & -dcos(\phi ) & 0 \end{array}\right]\dot{q}=0 \end{array}$$

In order to verify the holonomy of the previous mentioned system, the following definition is needed [71]:

**Definition 1.**  *Consider two vector fields given by* $f\in R^{n}$ *and* $g\in R^{n}$*, so the Lie brackets between these two vectors are given by:*

(3) $$\left[f,g\right]=\frac{\partial g}{\partial x}f-\frac{\partial f}{\partial x}g$$

*In which* $x={\left[x_{1},x_{2},\ldots ,x_{n}\right]}^{T}$ *in which:*

(4) $$\frac{\partial f}{\partial x}=\left(\begin{array}{ccc} \frac{\partial f_{1}}{\partial x_{1}} & \ldots & \frac{\partial f_{1}}{\partial x_{n}}\\ \vdots & \ddots & \vdots \\ \frac{\partial f_{n}}{\partial x_{1}} & \ldots & \frac{\partial f_{n}}{\partial x_{n}} \end{array}\right)$$

So, by defining the following vector fields:

(5) $$\begin{array}{ccc} g_{1}=\left(\begin{array}{c} 0\\ 0\\ 0\\ 1 \end{array}\right) & \; & g_{2}=\left(\begin{array}{c} -cos(\phi )\\ -sin(\phi )\\ -\frac{1}{d}\frac{sin(\theta )}{\cos(\theta )}\\ 0 \end{array}\right) \end{array}$$

Somehow, the Lie bracket of $g_{1}$ and $g_{2}$ is given by:

(6) $$\left[g_{1},g_{2}\right]=\left(\begin{array}{c} -sin(\phi )\\ -cos(\phi )\\ -\frac{1}{d}cos\left(\theta \right)sin\left(\phi \right)cotan\left(\phi \right) \end{array}\right)$$

So, the previous Lie bracket does not span the set of $g_{1}$ and $g_{2}$ and the system is non-holonomic [71].

## 5. Control Strategies Definitions

In this section, we define the two control strategies proposed in this research study. This section is divided into the following subsections in order to evince the main theoretical results:

- Neural controller definition.
- Driftless control strategy.
- Non-Driftless control strategy.

### 5.1. Neural Controller Structure

The neural controller structure consists of the following components:

(7) $${\tilde{y}}_{j}=\sum_{i=1}^{n}\left(w_{ji}\sigma \left(p+\theta_{i}\right)+\theta_{j}\right)$$

In which $w_{ji}$ represents the hidden unit weights of the neural network, $\theta_{i}$ represents the input weights, $\theta_{j}$ represents the bias of the neural network, and σ(.) is the activation function, which in this case is a sigmoidal function, and $p=\sum_{r=1}^{k}q_{r}$ for the inputs $q_{r}$ and the *j* output. The neural network (7) can be written in vector matrix form as follow:

(8) $$\begin{array}{ccc} \tilde{y} & = & \sum_{i=1}^{n}\underset{w_{i}}{\underbrace{\left[\begin{array}{c} w_{1i}\\ w_{2i} \end{array}\right]}}\sigma \left(p+\theta_{i}\right)+\underset{\theta }{\underbrace{\left[\begin{array}{c} \theta_{1}\\ \theta_{2} \end{array}\right]}}\\ \tilde{y} & = & \sum_{i=1}^{n}w_{i}\sigma \left(p+\theta_{i}\right)+\theta \end{array}$$

In which $w_{i}\in R^{2}$ is the hidden unit weights and $\theta \in R^{2}$ is the bias vector.

### 5.2. Driftless Control of the Mobile Robot

For the dynamic neural network control of the mobile robot, consider the following dynamic neural controller:

(9) $${\dot{x}}_{c}=K_{1}e+K_{2}x_{c}+\sum_{i=1}^{n}w_{i}\sigma \left(p+\theta_{i}\right)+\theta$$

In which $x_{c}\in R^{2}$ is the controller variable, $K_{1}\in R^{2\times 2}$ and $K_{2}\in R^{2\times 2}$ are the gain matrices and *p* is the neural network controller input. In order to establish the driftless control system, the following scheme is implemented:

(10) $$\dot{q}=g_{1}u_{1}+g_{2}u_{2}=\underset{G}{\underbrace{\left[\begin{array}{cc} g_{1} & g_{2} \end{array}\right]}}\underset{U}{\underbrace{\left[\begin{array}{c} u_{1}\\ u_{2} \end{array}\right]}}$$

In the following theorem, we define the driftless control law for the mobile robot:

**Theorem 1.**  *The driftless dynamic system (10) is stabilized by the following control law:*

(11) $$\begin{array}{ccc} U & = & -G^{-1}\frac{q}{{\alpha ∥q∥}^{2}}\eta x_{c}^{T}K_{1}e-G^{-1}\frac{q}{{\alpha ∥q∥}^{2}}\eta x_{c}^{T}K_{2}x_{c}\\ & - & \frac{q}{{\alpha ∥q∥}^{2}}\eta x_{c}^{T}\left[\sum_{i}^{n}w_{i}\sigma \left(p+\theta_{i}\right)+\theta \right]-G^{-1}q \end{array}$$

*in which* $\eta \in R^{+}$ *and* $\alpha \eta \in R^{+}$ *are positive gain constants and* $e=q_{d}-q$ *is the error variable in which* $q_{d}$ *is the desired trajectory of the mobile robot.*

**Proof.**  Consider the following Lyapunov function:

(12) $$V=\frac{\eta }{2}x_{c}^{T}x_{c}+\frac{\alpha }{2}q^{T}q$$

Obtaining the first time derivative of the previous Lyapunov function yields:

(13) $$\dot{V}=\eta x_{c}^{T}{\dot{x}}_{c}+\alpha q^{T}\dot{q}$$

Now, making the required substitution in the previous equation yields:

(14) $$\begin{array}{ccc} \dot{V} & = & \eta x_{c}^{T}K_{1}e+\eta x_{c}^{T}K_{2}x_{c}\\ & + & \eta x_{c}^{T}\left[\sum_{i}^{n}w_{i}\sigma \left(p+\theta_{i}\right)+\theta \right]+\alpha q^{T}GU \end{array}$$

So, making the required substitutions of (11) into the previous equation yields:

(15) $$\dot{V}=-\alpha q^{T}q<0$$

and the proof is completed. □

### 5.3. Non Driftless Control of the Mobile Robot

To achieve this, it is necessary to define the dynamic model of the mobile robot in the following way:

(16) $$\underset{G}{\underbrace{\left[\begin{array}{cccc} sin(\theta ) & -cos(\theta ) & 0 & 0\\ sin(\theta +\phi ) & -cos(\theta +\phi ) & -dcos(\phi ) & 0 \end{array}\right]}}\underset{\dot{q}}{\underbrace{\left[\begin{array}{c} \dot{x}\\ \dot{y}\\ \dot{\theta }\\ \dot{\phi } \end{array}\right]}}=\underset{U}{\underbrace{\left[\begin{array}{c} u_{1}\\ u_{2} \end{array}\right]}}$$

in which $U\in R^{2}$ is the control input. Now, consider the following neural dynamic controller:

(17) $${\dot{x}}_{c}=K_{1}e+K_{2}x_{c}+\sum_{i=1}^{n}w_{i}\sigma \left(p+\theta_{i}\right)+\theta$$

In which $K_{1}\in R^{4}$ and $K_{2}\in R^{4}$ are appropriate gain matrices. Meanwhile $\dot{e}={\dot{q}}_{d}-\dot{q}$ is the error dynamics and $q_{d}\in R^{4}$ is the desired trajectory of the mobile robot. The following theorem evinces how the dynamics of the mobile robot can be stabilized.

**Theorem 2.**  *The dynamic system of the mobile robot (16) is stabilized by the following control law as shown in:*

(18) $$\begin{array}{ccc} U & = & G{\dot{q}}_{d}-G\frac{e}{{\beta ∥e∥}^{2}}\rho x_{c}^{T}K_{1}e-G\frac{e}{{\beta ∥e∥}^{2}}\rho x_{c}^{T}K_{2}x_{c}\\ & - & G\frac{e}{{\beta ∥e∥}^{2}}\rho x_{c}^{T}\left[\sum_{i=1}^{n}w_{i}\sigma \left(p+\theta_{i}\right)+\theta \right]+Ge \end{array}$$

*In which* $\rho \in R^{+}$ *and* $\beta \in R^{+}$ *are appropriate control parameters.*

**Proof.**  Consider the following Lyapunov functional:

(19) $$V=\frac{\rho }{2}x_{c}^{T}x_{c}+\frac{\beta }{2}e^{T}e$$

So, taking the derivative of the previous Lyapunov function and making the appropriate substitutions yields:

(20) $$\begin{array}{ccc} \dot{V} & = & \rho x_{c}^{T}\left[K_{1}e+K_{2}x_{c}+\sum_{i=1}^{n}w_{i}\sigma \left(p+\theta_{i}\right)+\theta \right]\\ & + & \beta e^{T}\left[{\dot{q}}_{d}-G^{-1}U\right] \end{array}$$

Now by substituting (18) into the previous equation yields:

(21) $$\dot{V}=-\beta e^{T}e<0$$

So the system is globally stable, and the proof is complete. □

## 6. Numerical Experiments

In this section, two numerical experiments are performed to test and validate the theoretical results found in this research study. The numerical experiments conducted in this research study are intended to verify the following performance indicators:

- Minimization of the tracking error.
- Speed of response of the controller.
- Improvement in comparison with other control strategies.

The experiments performed in this research study are the following:

- Driftless control strategy.
- Non-driftless control strategy.

The conditions in which these experiments are performed is basically d=0.5 m taking into consideration that these control strategies are intended for kinematic control purposes.

### 6.1. Numerical Experiment 1

For this experiment, the following gain constants are implemented: $\eta =1\times 10^{-6}$, $\alpha =1\times 10^{-6}$. Now, consider the neural network parameters defined as:

(22) $$\begin{array}{ccc} W_{i} & = & \left[\begin{array}{cccc} 0.084825 & 0.087038 & 0.073776 & 0.098989 \end{array}\right]\\ \theta_{i} & = & \left[\begin{array}{cccc} 0.071581 & 0.096176 & 0.026009 & 0.086780\\ 0.048502 & 0.041201 & 0.083732 & 0.015706\\ 0.044176 & 0.037104 & 0.033042 & 0.024069\\ 0.081635 & 0.010157 & 0.035023 & 0.089209 \end{array}\right]\\ \theta & = & \left[\begin{array}{c} 0.080926\\ 0.066993\\ 0.044356\\ 0.046736 \end{array}\right] \end{array}$$

The experiment consists of driving the position variables *x* and *y* to the origin starting from an initial condition in order to obtain the maximum accuracy until the final desired value must be reached to obtain the maximum performance.

**Figure 2:**
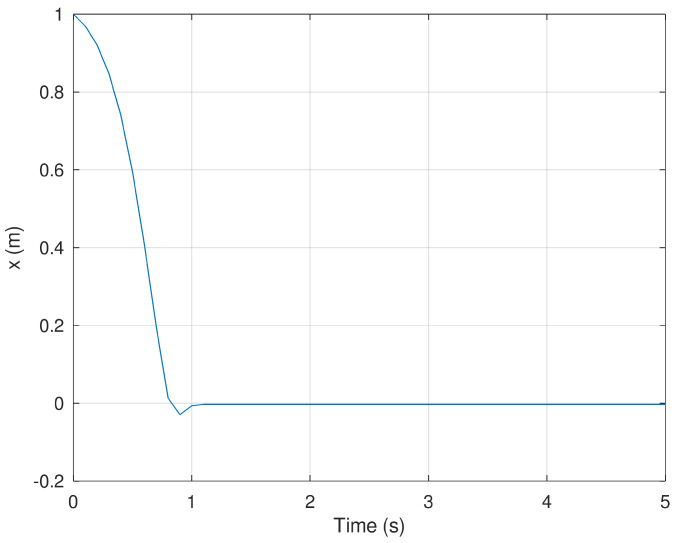
Position of the mobile robot in the *x* frame.

**Figure 3:**
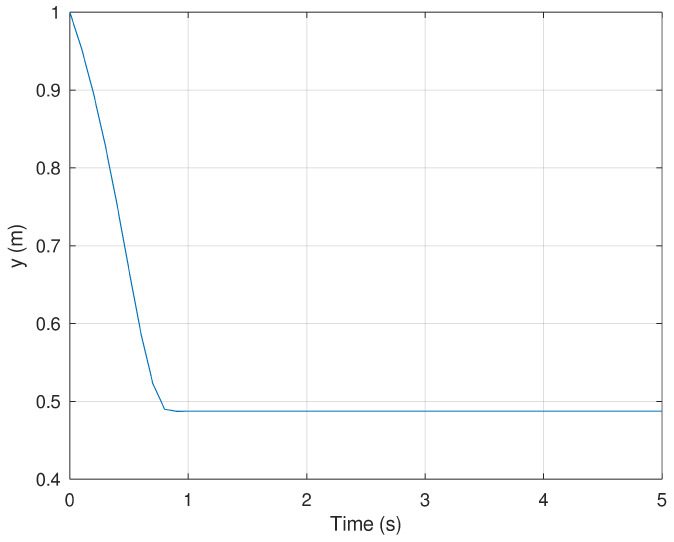
Position of the mobile robot in the *y* frame.

In Figure 2 and Figure 3 present the evolution in time of the mobile robot when it is driven from the initial condition to the origin in finite time. It is important to notice that the action of the controller drives these variables to the origin in approximately 1 s proving that the controller is effective despite the conditions in which the experiment is performed.

**Figure 4:**
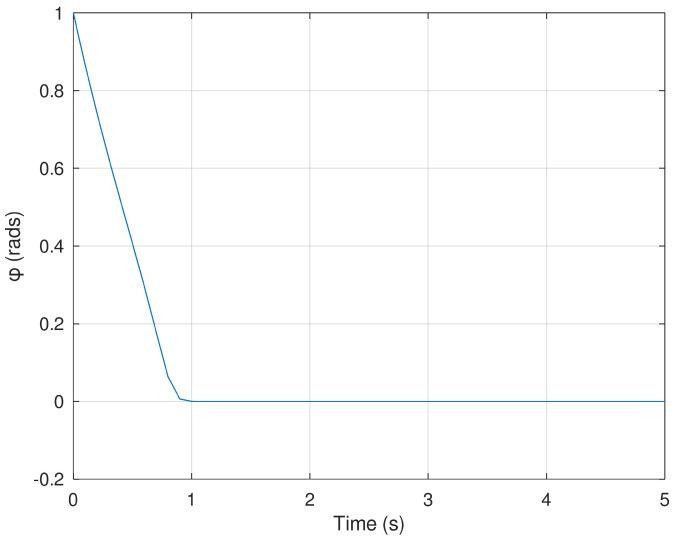
Evolution in time of the variable ϕ.

**Figure 5:**
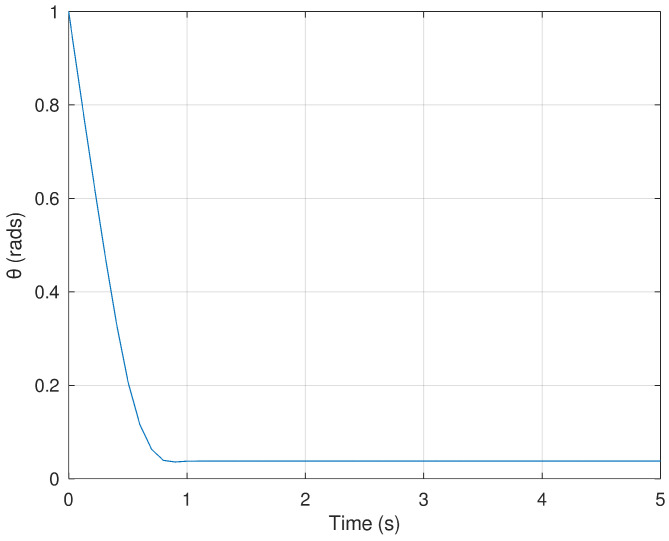
Evolution in time of the variable θ.

Meanwhile, Figure 4 and Figure 5 show the evolution in time of the angles of the mobile robot in order to drive the position variables *x* and *y* to the desired final value in finite time. The action of the neural controller and the dynamic surface controller are demonstrated to be fast and accurate in order to follow a predefined trajectory.

**Figure 6:**
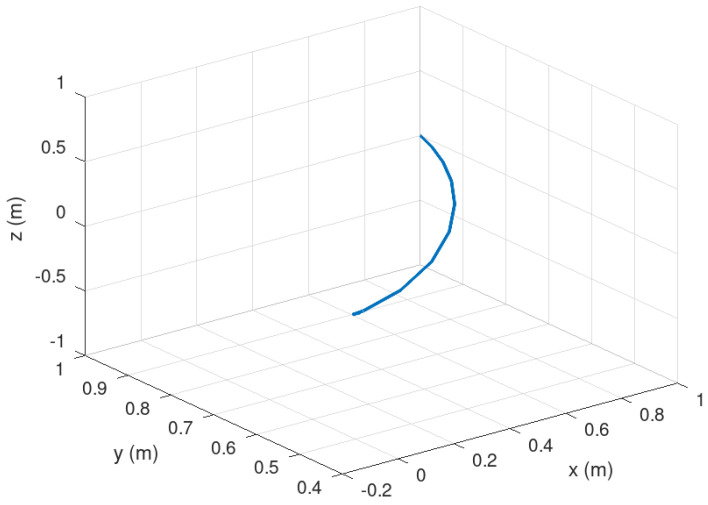
Evolution in time of the mobile robot trajectory.

Meanwhile, Figure 6 shows the trajectory of the mobile robot in 3D. It is corroborated in this figure how the trajectory is completed considering not only the position of the mobile robot but also the orientation of the mobile robot.

**Figure 7:**
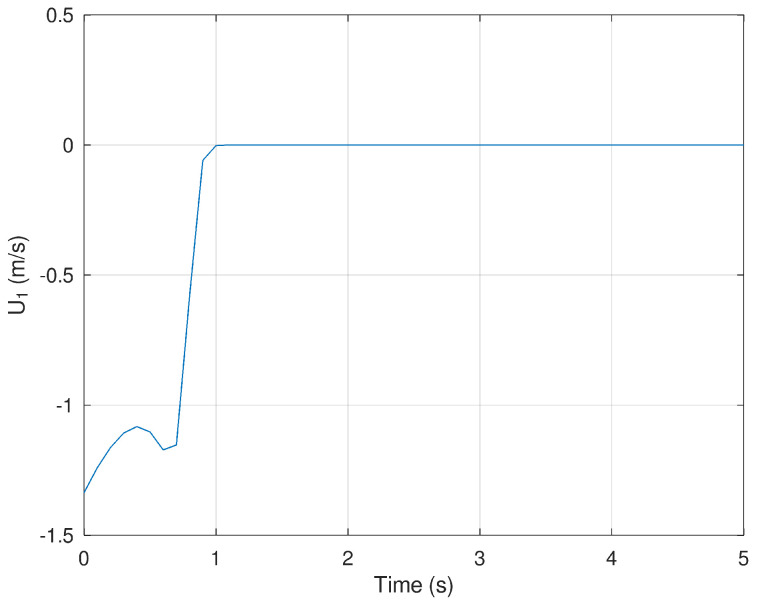
Evolution in time of the variable $U_{1}$.

**Figure 8:**
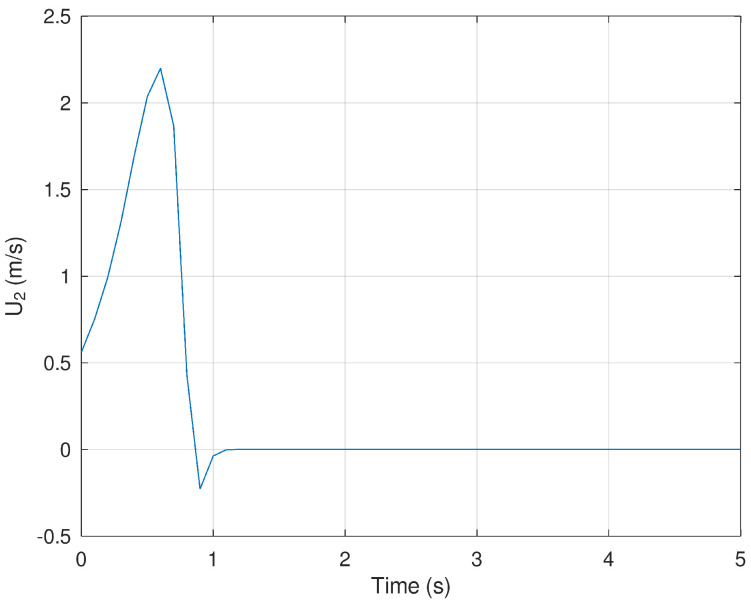
Evolution in time of the variable $U_{2}$.

Finally, Figure 7 and Figure 8 show the evolution in time of the control inputs $U_{1}$ and $U_{2}$. It is verified that a small control input is necessary to drive the position and orientation of the mobile robot in finite time.

### 6.2. Numerical Experiment 2

In this numerical experiment, we tested and validated the theoretical results regarding the synthesis of a non-driftless control of a mobile robot. This strategy, similar to the first experiment, consists of designing a neural-dynamic controller for trajectory-tracking purposes. In this experiment, the proposed control strategy is compared with the following strategies:

- Neural controller.
- Neural proportional-derivative PD controller.

The simulation parameters are the following; ρ=0.1, β=0.1, $K_{1}=1\times 10^{-1}$, $K_{2}=1\times 10^{-8}$. Meanwhile, the neural controller component has the following weights:

(23) $$\begin{array}{ccc} W_{i} & = & \left[\begin{array}{cccc} 1.1557\times 10^{-2} & 7.6815\times 10^{-3} & 7.8527\times 10^{-2} & 7.4791\times 10^{-2} \end{array}\right]\\ \theta_{i} & = & \left[\begin{array}{cccc} 0.083379 & 0.02538 & 0.051005 & 0.092401\\ 0.018102 & 0.090383 & 0.044346 & 0.067841\\ 0.063258 & 0.062726 & 0.079339 & 0.090871\\ 0.0421146 & 0.01078 & 0.006508 & 0.0038533 \end{array}\right]\\ \theta & = & \left[\begin{array}{c} 0.044756\\ 0.083565\\ 0.041632\\ 0.076618 \end{array}\right] \end{array}$$

**Figure 9:**
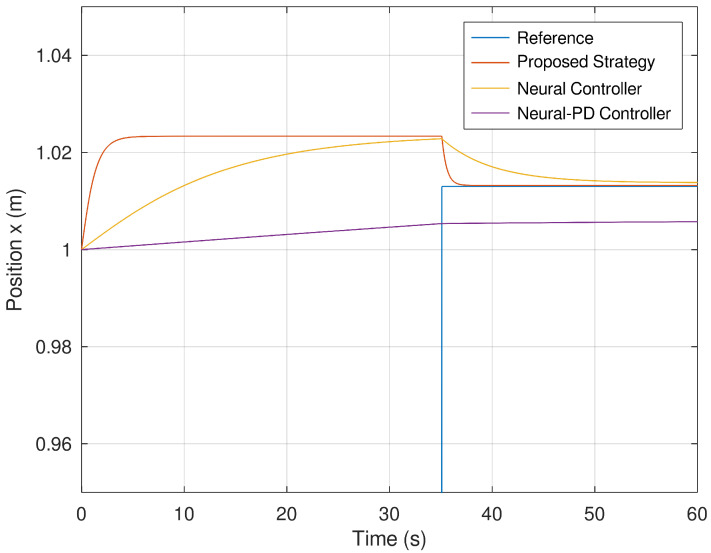
Position of the mobile robot in the *x* frame.

**Figure 10:**
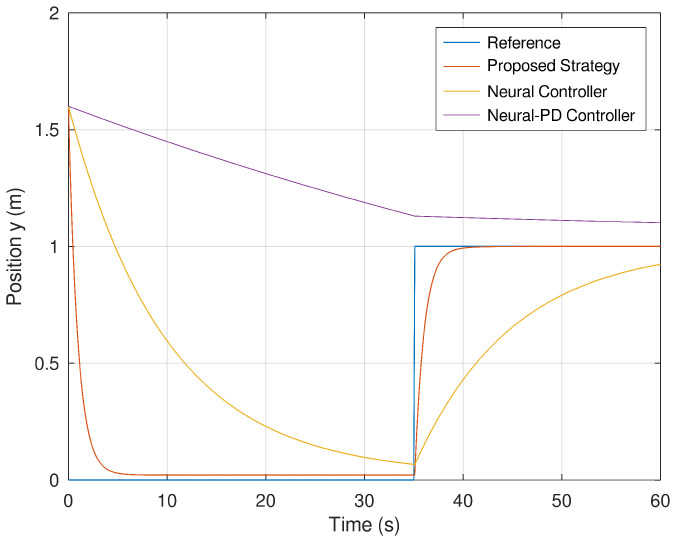
Position of the mobile robot in the *y* frame.

Figure 9 and Figure 10 show the evolution in time of the position variables *x* and *y* of the mobile robot. It is evinced that the controller synthetized with the proposed control strategy is more accurate in comparison with the neural and neural PD controllers. The proposed control strategy is more accurate in comparison with the strategies used as a comparative benchmark. The reason is because of the addition of a dynamic controller component that makes this control strategy more accurate and faster than the other control strategies.

**Figure 11:**
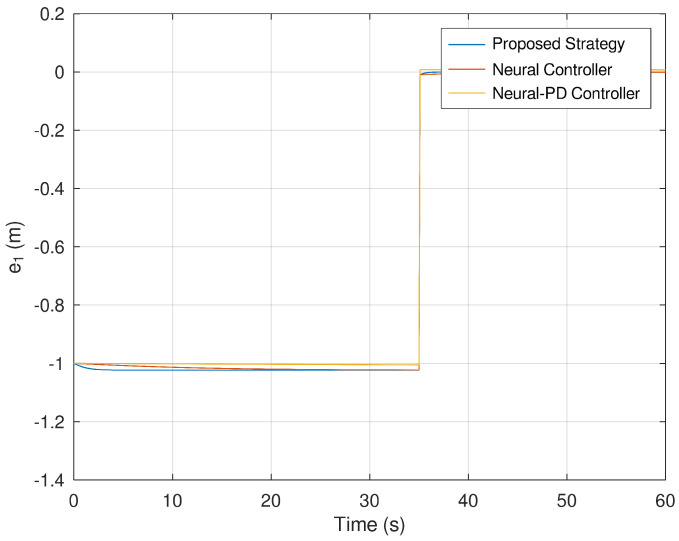
Evolution in time of the error variable $e_{1}$.

**Figure 12:**
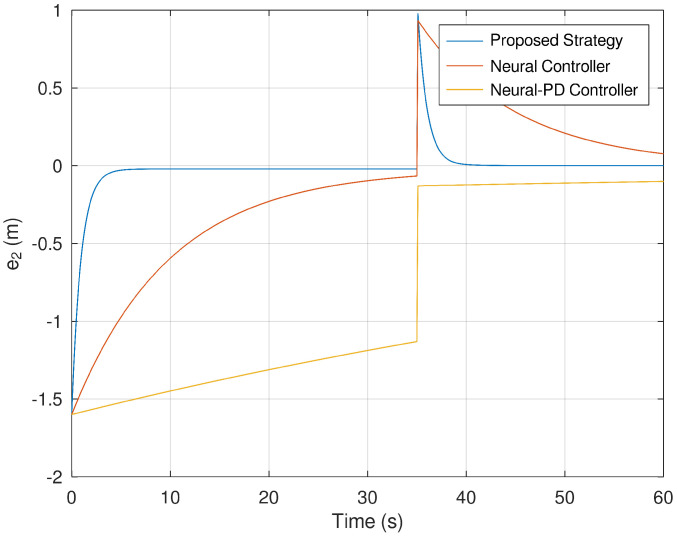
Evolution in time of the error variable $e_{2}$.

Meanwhile, Figure 11 and Figure 12 corroborate how the error variables reach the origin in finite time. As evinced in the previous figures, it is verified that the error variables yielded by the proposed control strategies reach the origin faster and more accurately than the neural and neural PD control strategies.

**Figure 13:**
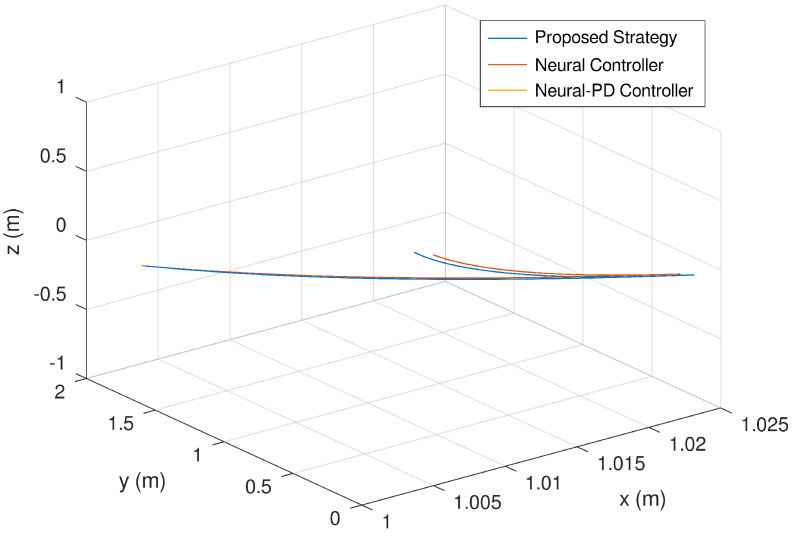
Evolution in time of the mobile robot trajectory.

Then, Figure 13 presents the trajectory of the mobile robot in 3D. It is evinced that the trajectory tracked by proposed controller is significantly better than the neural and neural PD controllers.

**Figure 14:**
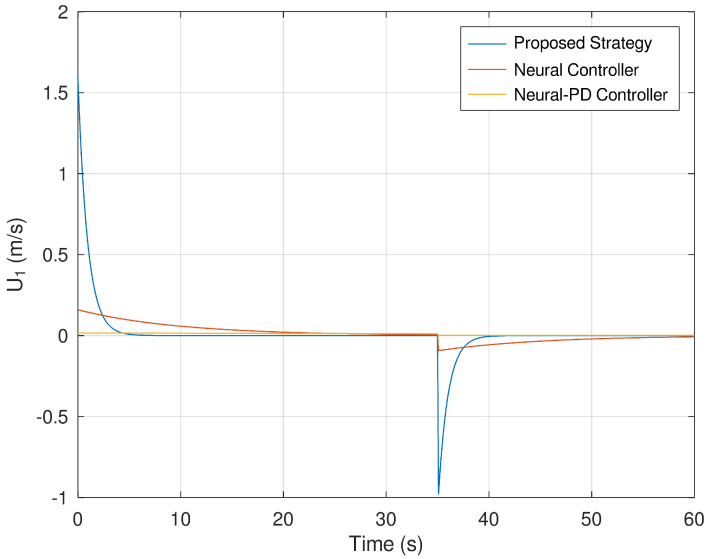
Evolution in time of the variable $U_{1}$.

**Figure 15:**
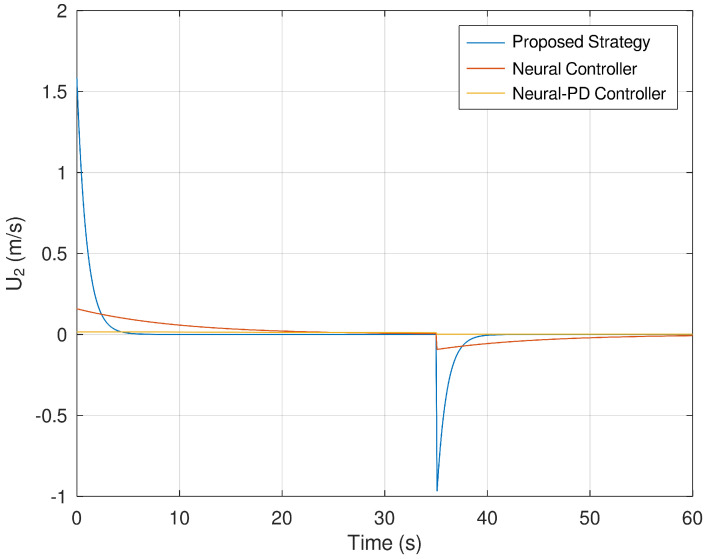
Evolution in time of the variable $U_{2}$.

Finally, Figure 14 and Figure 15 show the evolution in time of the control efforts for the variables $U_{1}$ and $U_{2}$. It is important to notice that the control effort for both control inputs is significantly smaller in comparison with the control effort generated by the neural and neural PD control strategies. This is an advantage when the controller is implemented in a real experimental setup. If the control effort is smaller, then unwanted effects like saturation are avoided.

## 7. Discussion

According to the theoretical and experimental results of this research paper, it is important to mention and discuss some important results and findings obtained. First, considering that the driftless control strategies with neural networks have not been reported extensively in the literature, in this research study, we proposed a combined neural network and dynamic controller for the trajectory tracking of mobile robots. We verified that the driftless control strategy is less costly in terms of control effort in comparison with other control strategies. In addition, the driftless control strategy presented in this research paper is useful for non-holonomic dynamics, which in this case is a mobile robot.

This paper demonstrates that the driftless control strategy provides an efficient mobile robot navigation strategy that is fast, reliable and accurate. It is worthwhile to mention that the neural network component of the proposed strategy provides an adequate control strategy that can be implemented and tuned relatively easily. The weights of the neural network do not need to be tuned offline by training methods like the Newton or Gaussian method, but it is important to remark that the neural networks used in this research study can be tuned by different optimization algorithms with less computational effort. The stable trajectory tracking of the mobile robot by implementing the neural network controller component ensured a precise stabilization by meeting the appropriate weight requirements, so several training and optimization algorithms can be implemented.

The dynamic controller part ensures that the stability of the driftless controller must be accurate and fast by meeting the adequate requirements of closed loop global stability. The controller was synthesized by selecting an appropriate Lyapunov function and obtaining the adequate control law. It is important to remark that the controller was carefully designed in order to be implemented in hardware easily, so the proposed control approach was relatively simple, considering also that the dynamics of the mobile robot is relatively simple but recognizing the complexity of non-holonomy of the mobile robot dynamics. This control strategy provides a smaller computational effort taking into consideration that the proposed controller is compact and the neural network controller only requires matricial and vectorial operations.

Meanwhile, the non-driftless controller is also reliable, accurate and fast, taking into consideration that this dynamic controller is more robust but requires slightly higher control effort than the driftless control system, but it is even more adequate for trajectory tracking in comparison with the driftless control system. It is important to notice that depending on the application of the mobile robot, one of these two control strategies is suitable. For this reason, in this research study, both strategies are investigated, taking into consideration that the control effort in some cases is required to be smaller to avoid some effects like saturation that can produce unwanted effects. In cases where the required hardware is available, the non-driftless control strategy is more adequate, so the driftless control strategy is suitable when the adequate hardware is not available.

## 8. Conclusions

This research paper presents a driftless and non-driftless control strategy based on neural dynamic controllers for the trajectory tracking of a mobile robot. Considering the non-holonomic characteristics of the mobile robot dynamics, an appropriate, relatively simple and implementable control strategy is provided in order for these results would be implementable in real-time hardware. The Lyapunov stability theorem is implemented for the design of the two control approaches in order to obtain a globally closed-loop stable system. Numerical examples validate the theoretical results obtained in this research study, proving that the theoretical results yield an appropriate performance of the mobile robot in order to track a predefined trajectory.

As a future direction, the next steps are to design a robust controller for the mobile robot considering different types of uncertainty for dynamic and kinematic control. Considering that uncertainties are found in real systems in practice, the dynamic modeling and design of a robust control strategy will be proposed. Besides, the consideration of disturbances and the implementation in a real experimental setup will be proposed in the future. The design of a novel disturbance rejection control will be also considered.

## Data Availability

No data was used in this research study.
